# Supplementary material for: Non-backtracking walks reveal compartments in sparse chromatin interaction networks
Source: Sci Rep. 2020 Jul 9;10:11398. doi: 10.1038/s41598-020-68182-0 (PMC7347895; doi:10.1038/s41598-020-68182-0)
Supplement: Supplementary file 1 — Supplementary information [file 41598_2020_68182_MOESM1_ESM.pdf]

# Non-backtracking walks reveal compartments in sparse chromatin interaction networks. Supplementary information

K. Polovnikov[1,2*], A. Gorsky[5,6], S. Nechaev[3,4], S. V. Razin[7,8], S. Ulyanov[7,8]

[1] *Institute for Medical Engineering and Science, Massachusetts Institute of Technology, Cambridge, MA 02139*

[2] *Skolkovo Institute of Science and Technology, 143026 Skolkovo, Russia*

[3] *Interdisciplinary Scientific Center Poncelet (ISCP), 119002, Moscow, Russia*

[4] *Lebedev Physical Institute RAS, 119991, Moscow, Russia*

[5] *Moscow Institute for Physics and Technology, Dolgoprudnyi, Russia*

[6] *Institute for Information Transmission Problems of RAS, Moscow, Russia*

[7] *Institute of Gene Biology, Russian Academy of Sciences, Moscow, Russia*

[8] *Faculty of Biology, M.V. Lomonosov Moscow State University, Moscow, Russia*

(Dated: May 20, 2020)

* To whom correspondence should be addressed. Email: kipolovnikov@gmail.com

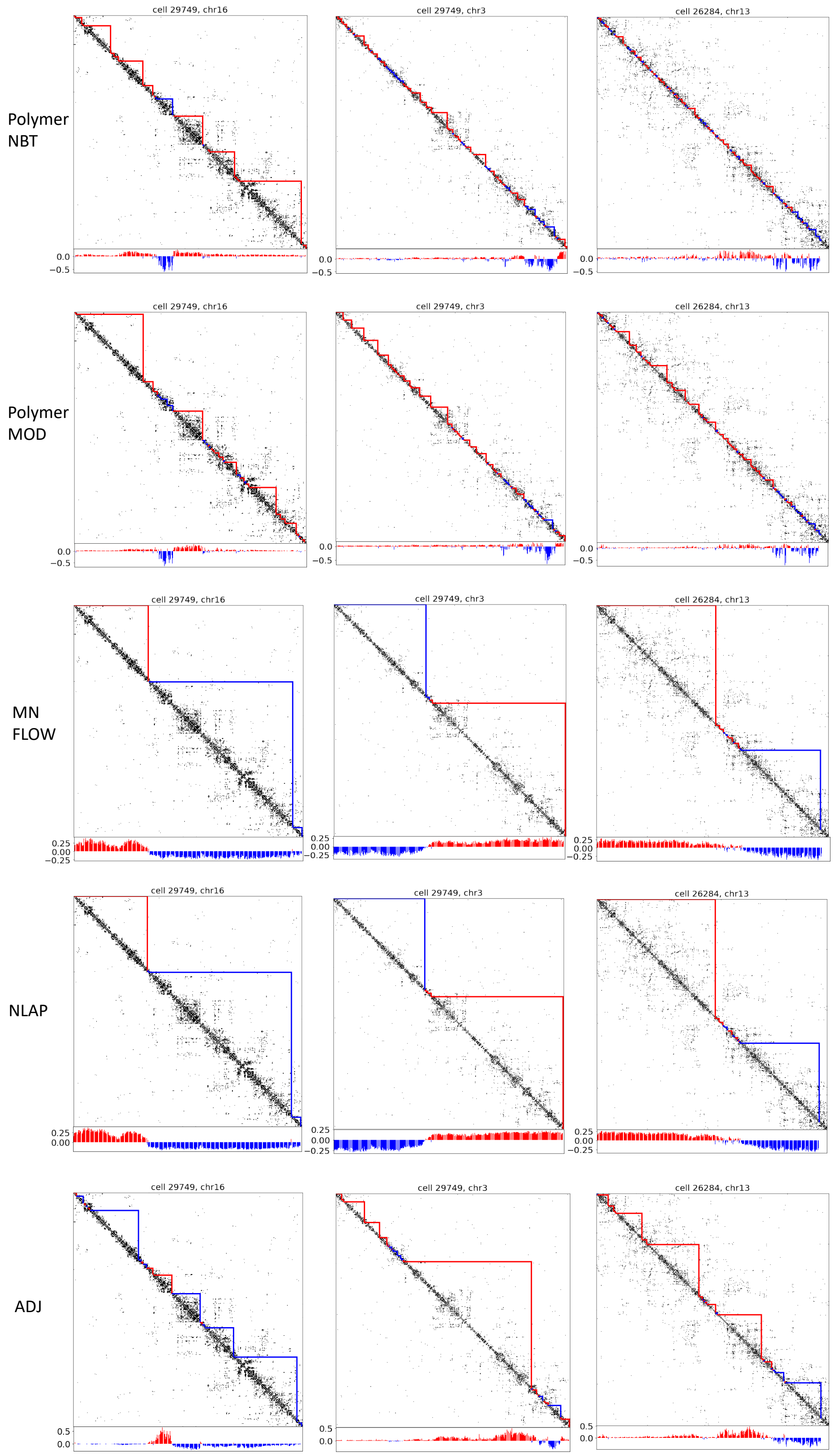


Figure S1: Annotations of active (red) and inactive (blue) compartmental domains for three chromosomes (16, 3 and 13; resolution 200kb) of the cell 29749 by the polymer non-backtracking flow operator, polymer modularity, M. Newman's non-backtracking flow, normalized Laplacian and adjacency. Below each map the compartmental signal from the leading eigenvector of the corresponding operator is provided. Hi-C data is taken from Flyamer et al.

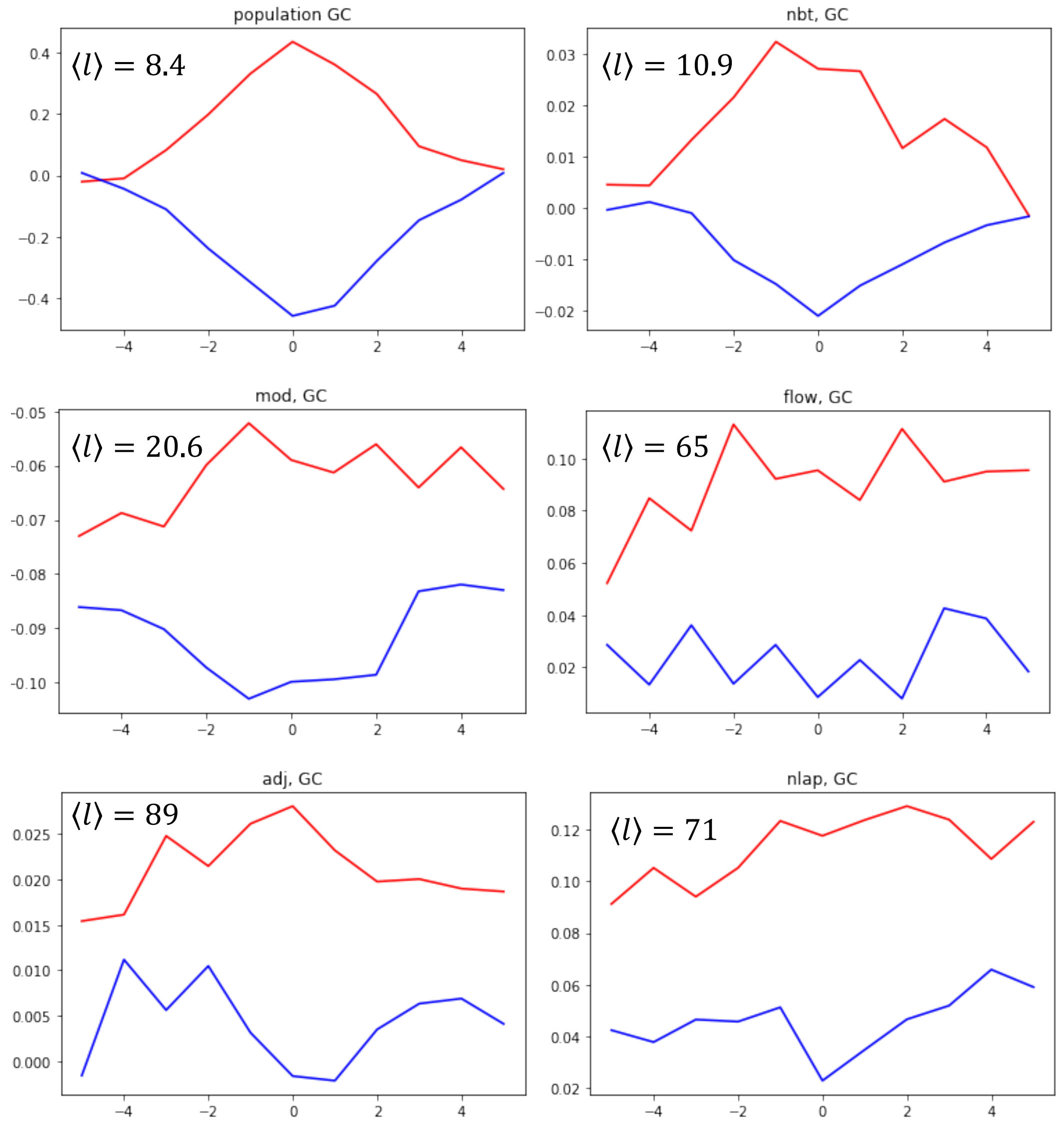


Figure S2: Averaged profiles of the GC content (z-scores) plotted around the centers of the compartmental domains (active - red, inactive - blue) for the population (embryonic stem cells, data taken from Bonev et al.), polymer non-backtracking flow operator, polymer modularity, M. Newman's non-backtracking flow, normalized Laplacian and adjacency. In case of single cells the average is taken over all compartmental domains of respective type from 260 contact maps. Mean sizes of the domains in bins (200 kb), inferred by each operator, are labeled on the plots.

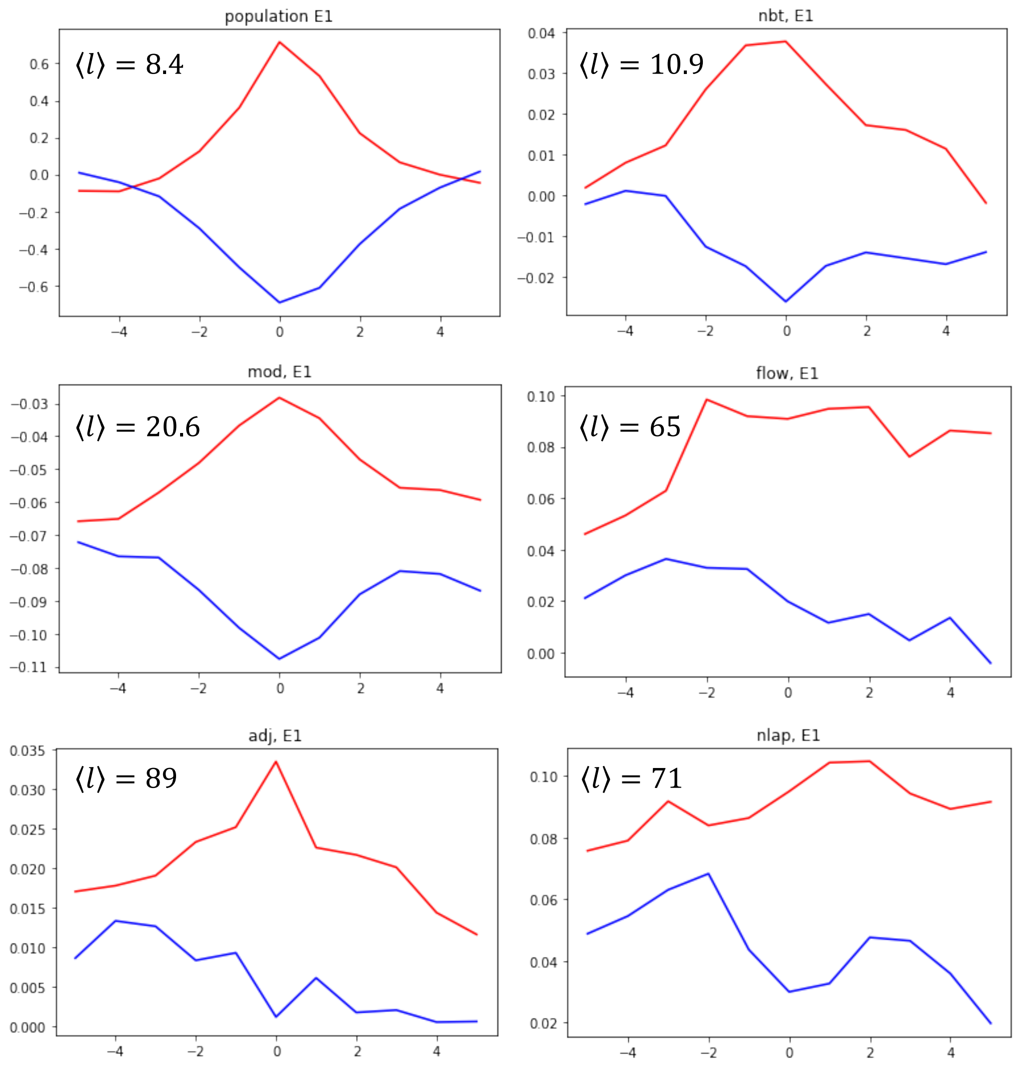


Figure S3: Averaged profiles of the leading eigenvector of the population-averaged Hi-C map (z-scores, ES cells, data taken from Bonev et al.) plotted around the centers of the compartmental domains (active - red, inactive - blue). The bulk matrices are preliminary normalized over expected and the eigenvector is phased with respect to the GC content, as usual. The profiles are demonstrated for the same population (ES cells) and for the domains determined in single cells by means of the polymer non-backtracking flow operator, polymer modularity, M. Newman's non-backtracking flow, normalized Laplacian and adjacency. In case of single cells the average is taken over all compartmental domains of respective type from 260 contact maps. Mean sizes of the domains in bins (200 kb), inferred by each operator, are labeled on the plots.

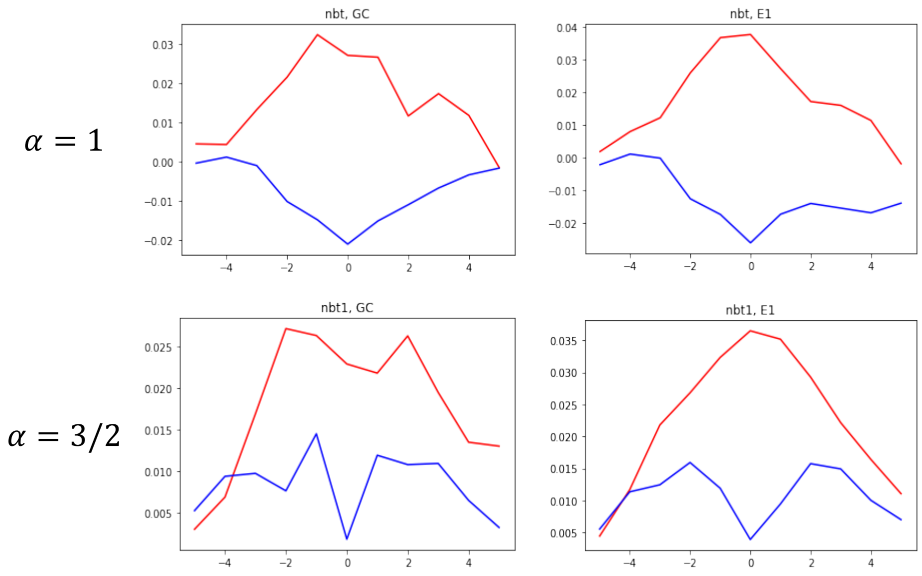


Figure S4: Comparison of the compartmental domains, inferred by the polymer non-backtracking flow for two values of $\alpha = 1$ (fractal globule) and $\alpha = 3/2$ (ideal chain). The profiles for the GC content and for the bulk leading eigenvector E1 are demonstrated.
